# The impact of physical fitness, social life, and cognitive functions on work ability in middle-aged and older adults

**DOI:** 10.1007/s00420-022-01943-8

**Published:** 2022-12-16

**Authors:** Jennifer A. Rieker, Patrick D. Gajewski, José Manuel Reales, Soledad Ballesteros, Klaus Golka, Jan G. Hengstler, Edmund Wascher, Stephan Getzmann

**Affiliations:** 1 Department of Basic Psychology II, Universidad Nacional de Educación a Distancia, Juan del Rosal, 10, 28040 Madrid, Spain; 2 Leibniz Research Centre for Working Environment and Human Factors (IfADo) at the Technical University of Dortmund, Dortmund, Germany; 3 Department of Methodology for Behavioral Science, Universidad Nacional de Educación a Distancia, Madrid, Spain

**Keywords:** Work ability index, Aging, Occupational health, Lifestyle factors, cognition, structural equation model (SEM)

## Abstract

**Objective:**

Demographic changes encompass societies to maintain the work ability (WA) of aging workforces. The present study explored the relationship between modifiable lifestyle factors, cognitive functions, and their influence on WA, using a multi-group structural equation approach.

**Method:**

Cross-sectional data from 247 middle-aged and 236 older employees from the Dortmund Vital Study were included in this analysis. We proposed a model with three exogenous variables (Physical Fitness, Cognitive Functions, and Social Life), and with WA as the endogenous variable. WA was measured with the Work Ability Index (WAI), which considers job demands and individual physical and mental resources. Multi-group analyses were based on the principles of invariance testing and conducted using robust estimation methods.

**Results:**

Results revealed that Social Life outside work had significant positive effects on WA in both, middle-aged and older adults. Physical Fitness had a significant effect on WA only in middle-aged adult, and Cognitive Functions had no significant influence on WA in either group. In older adults, Physical Fitness correlated with Cognitive Functions, whereas in middle-aged adults, Cognitive Functions marginally correlated with Social Life.

**Conclusions:**

Our results underline the importance of an active social life outside the workplace for WA, regardless of the employees’ age. The influence of Physical Fitness on WA changes with increasing age, indicating the necessity to have a differentiated view of age effects and interacting influencing factors. Our research contributes to the knowledge of how WA could be most effectively promoted in different age groups.

Clinicaltrials.gov NCT05155397; https://clinicaltrials.gov/ct2/show/NCT05155397.

**Supplementary Information:**

The online version contains supplementary material available at 10.1007/s00420-022-01943-8.

## Introduction

Western population is aging rapidly due to increasing life expectancy and decreasing fertility rates, confronting societies with new challenges. It was estimated that in the European Union, the old-age dependency ratio (people aged 65 and above relative to those aged 15 to 64) will increase from 29.6% in 2016 to 51.2% in 2070 (European Commission 2018). Aging affects the macroeconomic structures in two ways: First, public health care is mainly financed by social security contributions of the working population, and the health care expenditure largely depends on the health status of the retired population. Thus, demographic changes confront societies to balance public spending on pensions and health care versus the need to reduce budget deficits (Harper 2014). Second, population aging comes along together with the aging of the working population, a phenomenon referred to as “workforce aging” (Aiyar et al. 2016). Workforce aging directly influences the labour productivity levels, which increase up to the age of 50 years and decrease toward the end of the working life (Aiyar et al. 2016). 

Workforce aging is closely related to work ability (WA), which is defined as the balance of physical, mental, and social abilities on the one hand, and work conditions and requirements on the other hand (Gould et al. 2008). A widely used measure of WA is the Work Ability Index (WAI; Ilmarinen 2007), an instrument often used in Occupational Safety and Health. Lower levels of WA have been related to a longer duration of sickness absence, early retirements, and even with increased risk of premature death (Bethge et al. 2021). WA is a multifactorial concept that is influenced by work-related factors, such as work demands and resources, expertise, attitudes toward work, and individual factors, such as health, age, and psychosocial factors (Gould et al. 2008; van den Berg et al. 2008a). Within the individual antecedents of WA, physical activity has been repeatedly related to improved WA (Airila et al. 2012; Mohammadi et al. 2014), especially when the exercise has moderate-to-high intensity (Calatayud et al. 2015; Grabara et al. 2018; van den Berg et al. 2008a), and in workers with high mental work demands (Prieske et al. 2021).

Furthermore, social support, as well as adaptive social functioning have been related to higher WA (Brady et al. 2020; Gould et al. 2008; Peters et al. 2018). A recent review on interventions to promote WA at workplaces (Lusa et al. 2020) found that the most effective interventions were group-based actions, such as stress management, or remote counselling, within others, stressing out the importance of social support to WA. Peters et al. (2018) reported that social support, operationalized by the number of contacts in case of problems, the amount of interest and participation of other people, and help of the neighbourhood in providing practical assistance, significantly predicted WA. This relationship held even when controlling for sociodemographic and work-related factors, as well as for personality traits.

An important component included in the measure of WA are mental resources. However, even though the WAI includes items that relate to psychiatric diseases such as depressive symptoms or anxiety, and to motivational or emotional factors such as general activity or confidence, none of the WAI dimensions are related to cognitive functioning. Cognitive functions refer to different mental processes involved in perceiving, attending, learning, maintaining, and manipulating information that enable goal-directed behavior and may reflect crucial preconditions of successful WA in several occupational areas (Gajewski and Falkenstein 2011). Whereas some studies reported no significant relationship between cognitive variables and WA (Eskelinen et al. 1991), others found a significant relationship between WA and the cognitive status (Chung et al. 2015), or a relationship only in lower performing older adults (Gould et al. 2008). Normal aging is associated with a decline in several cognitive functions (Park and Reuter-Lorenz 2009; Reuter-Lorenz and Park 2014; Salthouse 2012), which might affect a worker’s ability to acquire new knowledge and skills necessary to adapt to changing work requirements (Salthouse 2012), leading to a decrease in motivation at work (Fisher et al. 2019) and earlier retirement (Belbase et al. 2015).

Therefore, there is a need to maintain or even improve cognitive abilities in middle-aged and older employees to accomplish current and future work demands and to be able to adjust to new work demands, especially in mentally demanding jobs. Several factors have been related to improved cognitive functioning in later life, such as engagement in social activity and large social networks (Evans et al. 2019; Kelly et al. 2017), cognitive training, and physical exercise (Ballesteros et al. 2015, 2018; Gajewski and Falkenstein 2016; 2018; Gajewski et al. 2017; Rieker et al. 2022). Overall, previous findings suggest a relationship between physical, cognitive, and social aspects on the one hand and WA on the other. However, the strength of the respective correlations, as well as possible dependencies on the age of the employees, are still poorly understood. 

From a point of view of occupational health, the antecedents of WA are based on a balance between a person’s resources and work demands (Ilmarinen 2001). The idea behind WA is often depicted as a “house”, with four floors and its nearby environment representing five interrelated dimensions sustaining WA (Gould et al. 2008; Ilmarinen et al. 2005). The first three floors represent an individual’s personal resources, with functional capacities as the first floor, occupational competence as the second floor, and attitudes and motivations as the third floor. The fourth floor is represented by demands of the work, such as working conditions, organization of work, work community, and management. Two buildings in the surroundings represent relations between work, family and close community, and leisure activities. Analyses of the convergent and discriminant validity of the model showed that health, functional capacities, and leisure activities showed unique convergence with different WA assessment tools, highlighting their importance as the foundation of the WA “house” model (Ilmarinen 2015). Furthermore, this model has proven to be invariant across gender and partially invariant across age and employee groups (Ilmarinen 2015). The testing of model invariance is a commonly used statistical method to investigate group differences by means of multi-group confirmatory factor analyses (Marcoulides and Heck 1993; for an updated guideline using Mplus and lavaan, see Svetina et al. 2020). The analysis typically begins with fitting the data of two groups simultaneously into two models: one in which all parameters are allowed to differ between groups, and one in which all parameters are fixed to those obtained from analysis of the pooled data across groups. If the two models are not significantly different, and the latter fits the data well, then one can assume that there is no variation in the path coefficients by group and a multi-group approach is not necessary. If they are, then the next step would be to explore which paths are the same and which are different. This is achieved by sequentially constraining the coefficients of each path and re-fitting the model. 

The contribution of physical activity, cognitive functioning, and social life outside work to WA has been corroborated by numerous investigations (Gould et al. 2008; Nye et al. 2022; Peters 2018; van den Berg et al. 2008a, b). However, the differential effect of these factors on WA as a function of age remains largely unexplored. Physical and mental abilities are probably the personal resources that mostly change with age, thus affecting WA differently across the lifespan (Ilmarinen 2001; Nygård et al. 1991). By contrast, the positive influence of social functioning might be rather stable, as social networks are mostly fostered over the years and sustained by stable personality traits (Asendorpf and Wilpers 1998). Furthermore, when a specific personal resource declines with age, the individual might recruit, or optimize alternative resources to meet the job demands. The dynamic between a person’s abilities and job demands is represented in the WA model by the free intercorrelations between its dimensions. As each of the dimensions could potentially buffer, or compensate for the low functioning of the others, the WA house provides a useful framework for analyzing the theoretical antecedents of WA within a multi-group approach of different age groups.

The main objective of the present study is to broaden our knowledge of the differential contribution of the components that compose the basement floor (physical fitness and cognitive functions) and surroundings (social life) of the WA house model. In this line, we aim to analyze if the respective effects of physical fitness, social life, and cognitive functions on WA, and their intercorrelations are invariant in middle-aged and older adults, or if their respective contributions to WA change across age. 

### Physical fitness

The contribution of health to WA is assessed within the WAI with three items that relate to physical diseases and sick leaves. Despite the normal biological decline in health with aging, older adults might manage to compensate for this decline and suffer fewer diseases with higher physical fitness levels. Thus, we expect to find a major influence of physical fitness on WA in older than in middle-aged adults.

### Cognitive functioning

Cognitive ability was found in previous research to be a consistent predictor of job performance (Nye et al. 2022; Schmidt and Hunter 1998). Cognitive ability declines with age (Reuter-Lorenz and Park 2014; Salthouse 2012); however, findings on differences in job performance between younger and older workers reveal only minimal differences (Ng and Feldman 2008), which appears paradoxical, especially in jobs with a high mental workload. Thus, a minor contribution of cognitive functioning to WA in older adults together with stronger relationships between other model parameters could be indicative of a compensatory mechanism to maintain WA. Previous studies have shown that physical exercise promotes neuroplasticity (Rasmussen et al. 2009; Walsh and Tschakovsky 2018), leading to cognitive improvements in older adults (Gajewski and Falkenstein 2016; Muiños & Ballesteros 2018). Furthermore, the link between exercise and cognition is age-dependent, with stronger effects when cognitive functions are at their decline than when they are at their peak efficiency.

### Social life

Previous work has shown that social life outside work constitutes an important antecedent of WA. Healthy social interactions are closely related to the reinforcement of a worker’s self-esteem (Bénabou & Tirole 2002), to a worker’s career adaptability (Wang and Fu 2015), and ultimately enable individuals to fully participate in life’s opportunities for growth and development (Feeney and Collins 2015). In older adults, interventional studies on cognitive and physical training have shown higher training gains when the training is performed in a social context (Rieker et al. 2022), highlighting the importance of social interactions as a motivational factor. For these reasons, we expect social life not only to significantly contribute to WA, but also to be related to more physical fitness and better cognitive functioning.

**Fig. 1:**
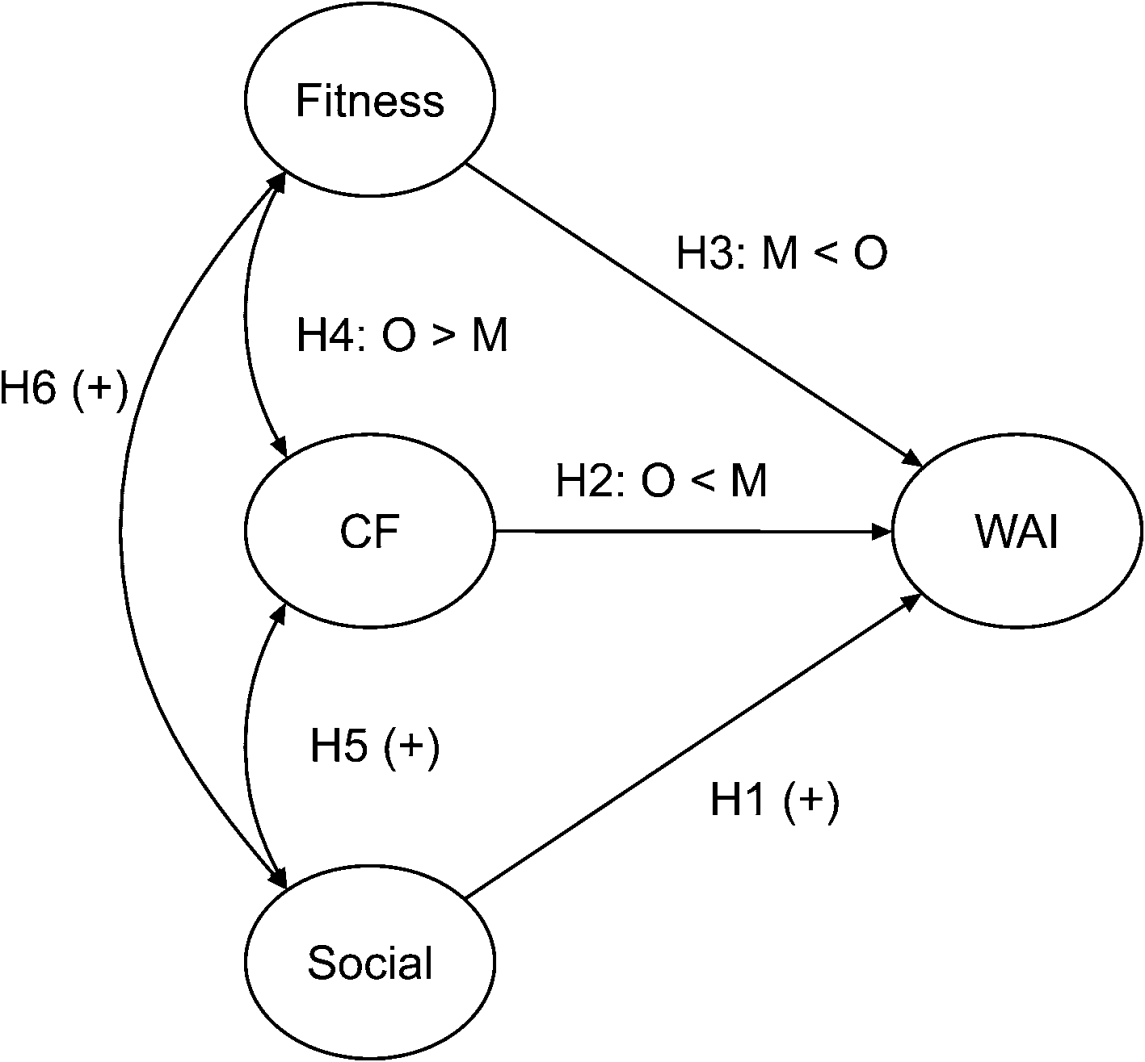
Hypothesized model. One-headed lines represent unstandardized regression coefficients, and two-headed curved lines represent correlations. *O* older adults, *M* middle-aged adults, *Social* social life, *CF* cognitive functions, *WAI* work ability index

Taken together, our working hypotheses are as follows (see Fig. 1):

- Hypothesis 1: Social Life will contribute significantly to WA in both groups.
- Hypothesis 2: Cognitive Functions will contribute less to WA in older adults than in middle-aged adults.
- Hypothesis 3: Physical Fitness will contribute less to WA in middle-aged adults than in older adults.
- Hypothesis 4: Cognitive Functions will correlate stronger with Physical Fitness in older than in middle-aged adults.
- Hypothesis 5: Social Life will correlate with Cognitive Functions in both groups.
- Hypothesis 6: Social Life will correlate with Physical Fitness in both groups.

## Method

### Participants

The present study used data from a subsample of the *Dortmund Vital Study* (for a detailed description of the study protocol, see Gajewski et al. 2022). Participants were recruited via an internet site, newspaper advertisements, reports and announcements in local print and radio media, public information events, social media, and flyers throughout the city of Dortmund, Germany. From the total number of 588 participants, we selected participants that were currently employed in part- or full-time jobs and had completed the long version of the Work Ability Index (WAI). The final sample comprised 483 healthy adults (63% women), aged 20 to 69 years (*M *_*age*_ = 42.66, SD = 12.76). For detailed information on participants’ sociodemographic and lifestyle characteristics, see Online Resource Table S1. With the objective to analyze the data across age groups, the participants were split into two groups, based on the median age: those in the age range of 20 to 44 years (64% women and 36% men) comprised the middle-aged group (*M*_*age*_ = 31.78, SD = 6.71), while those from 45 years on (63% woman) comprised the older aged group (*M*_*age*_ = 54.05, SD = 5.68). Selection criteria were full or part-time employment, a complete Work Ability Index (Ilmarinen 2007), no current history of psychiatric or neurological pathology, a score of 26 or above on the Mini-Mental State Examination (MMSE, Folstein et al. 1975), and a maximum score of 28 (no to moderate depression) on the Becks Depression Inventory II (Beck et al. 1996).

### Measures

WA was assessed with the WAI (Ilmarinen 2007), which considers the workers’ self-assessed physical and mental capacity in relation to work requirements, health status, and the worker resources. The questionnaire consists of seven self-reported parts with higher scores indicating better adjustment: (WAI1) “subjective estimation of current work ability compared with lifetime best”, with scores ranging from 0 to 10; (WAI2) “subjective work ability in relation to job demands”, with scores ranging from 2 to 10; (WAI3) “number of current diseases diagnosed by a physician”, with five categories ranging from 1 to 7; (WAI4) “subjective estimation of work impairment due to diseases”, with six categories scores ranging from 1 to 6; (WAI5) “sick leave during the past year”, with five categories ranging from 1 to 5; (WAI6) “own prognosis of work ability two years from now”, with three categories ranging from 1 to 7; and (WAI7) “mental resources”, with four categories ranging from 1 to 4.

Cognitive functions were evaluated with a series of paper-and-pencil psychometric tests that covered the most relevant work-related cognitive functions within the following categories: (a) Stroop Test, measuring interference control and inhibition; (b) Digit-Span Backward (DS) (Wechsler 1997), measuring working memory; (c) Performance Testing System (Horn 1962), measuring logical reasoning (LPS3) and spatial rotation (LPS7); (d) d2-R (d2) (Brickenkamp et al. 2010), measuring selective attention and attentional endurance; (e) Digit-Symbol-Test (DST) (Wechsler 1997), measuring processing speed.

Physical fitness was assessed with two parameters derived from the Physical Work Capacity Cycle Test (PWC-130), which evaluates the power output at a projected heart rate of 130 beats per minute: (a) the power-to-weight ratio (watt/kg) (PWR), and (b) the maximum power output in watt (Pmax). Furthermore, the duration of weekly physical activity was estimated with the Lüdenscheid Physical Activity Questionnaire [(LPAQ), Höltke and Jakob 2002]. This questionnaire consists of 13 questions and provides an assessment of weekly physical activity in the last 2 years, such as walking, cycling, jogging, swimming, dancing, gardening, or other sports.

Social Life was assessed with (a) the domain “Social relationships” of the WHO Quality of Life questionnaire (QoLsoc) (WHOQoL Group 1994). This domain is made up of three items assessing personal relationships, social support, and sexual activity. The items are rated on a 5-point scale with a maximum domain score of 20. Furthermore, a non-standardized sociodemographic questionnaire assessed (b) the number of friends (FriendsN), and (c) the frequency of social meetings (FriendsFreq). These items were assessed on a 5-point scale, ranging from zero (none/never) to five (8 or more/more than three times per week).

A detailed description of the assessment tools can be found in the study protocol of the *Dortmund Vital Study* (Gajewski et al. 2022).

### Data analysis

The analysis of missing data (4.43%) revealed no systematic pattern of missingness, and missing data were handled with pairwise deletion. Items with empty categories in one of the groups were recoded by collapsing the empty category/ies with the subsequent category. For example, none of the middle-aged scored in the first and third category of the ten categories of WAI6, whereas there were six cases in older adults. By this, WAI1 passed from the original ten categories to eight, WAI2 from eight to six, WAI4 from six to four, and WAI6 from three to two. We inversed the time scores of the Stroop test, to keep the same direction as in the other variables (the more the better). Correlations were assessed by means of heterogenic correlations (polychoric, polyserial, and Pearson) as required by the variables’ original scale types.

The hypothesized model comprised three exogenous factors (Physical Fitness, Cognitive Functions, and Social Life) and one endogenous factor (Work Ability). Work Ability was measured with seven items of the Work Ability Index (see “Measures” for a detailed item description). Physical Fitness was measured with three continuous variables (PWR, Pmax, and LPAQ), Cognitive Functions with six continuous variables (Stroop, d2, LPS3, LPS7, DST, and DS), and Social Life with one continuous variable (QoLsoc) and two ordinal variables (FriendsN, and FriendsFreq). We evaluated the model in the following steps: (1) we verified the model for the pooled data of both groups; (2) we verified the model in a multi-group approach by establishing configural and metric invariance, and (3) we compared regression coefficients and correlations between latent factors across groups. In a configural invariant model, parameters of both groups are allowed to freely vary, and acceptable fit indices determine that the covariance matrices of both groups can be fitted by the same factor model (Millsap and Olivera‑Aguilar 2012). In a metric invariant model, factor loadings are set to be equal across groups, and acceptable fit indices determine that the latent constructs are defined similarly by both groups (Millsap and Olivera‑Aguilar 2012). Statistically, we evaluated the differences in model fits with the difference in the comparative fit index (CFI) (Hu and Bentler 1999) and the difference of chi-square across nested models. If the difference in CFI is less than 0.01, the difference in model fit is considered trivial (Cheung and Rensvold 1999). In absence of multivariate normality, and to consider the ordinal metrics of the factor indicators, we used the weighted least square mean and variance adjusted (WLSMV) estimator for the structural equation modelling. For model identification, we fixed the first loading of each factor to one, and the latent intercepts (mean) of the first group to zero. The global goodness of fit of the models was evaluated with the root-mean-square error of approximation (RMSEA), the CFI, and the Standardized Root Mean Square Residual (SRMR). A CFI above 0.95, an RMSEA below 0.06, and a SRMR below 0.05 indicate an excellent fit, whereas a CFI above 0.90, an RMSEA and SRMR below 0.08 indicate an adequate fit. Statistical analysis was performed using the IBM SPSS v. 28.0 statistical software and the *lavaan* package (version 0.6–9) (Rosseel 2012) within the R software environment (version 4.1) (Core Team 2021). Figures were depicted using the packages *semPlot* (version 1.1.4) (Epskamp 2015) and *qgraph* (version 1.9) (Epskamp et al. 2012) for R.

## Results

### Descriptive statistics

Table 1 shows the descriptive statistics of sociodemographic and health-related variables, and the variables used in the structural equation modelling for middle-aged and older adults. Middle-aged adults showed overall higher levels in health-related variables (body mass index, waist–hip ratio, HRmax) and global cognition (MMSE). They also scored higher in education than older adults, which might be explained by more access to higher education in younger generations. Participants of both groups performed predominantly mental work as compared to physical, or mixed mental–physical work.

**Table 1:** Descriptive statistics of sociodemographic and health-related variables, and of the variables used in structural equation model for middle-aged and older adults

	Mean or frequency (SD or %)		*t* or ${\chi }^{2}$
	Middle-aged	Older	
*N*	247	236	0.251
Age	31.78 (6.71)	54.05 (5.68)	− 39.281***
Men	90 (36.4)	87 (36.9)	0.051
Woman	157 (63.6)	149 (63.1)	0.209
Education			
Junior high school or less	2 (0.8)	19 (8.1)	13.762***
Senior high school	18 (7.4)	19 (8.1)	35.787***
High school diploma	94 (38.7)	57 (24.2)	9.066**
University or higher	129 (53.1)	84 (35.6)	9.507**
Type of work:			
Physical work	17 (7.1)	12 (5.1)	0.862
Mental work	161 (66.6)	142 (60.4)	1.191
Mixed mental and physical work	63 (26.1)	81 (34.1)	2.25
BMI	24.89 (4.69)	26.06 (4.58)	− 2.779**
WHR	0.89 (0.05)	0.91 (0.06)	− 2.774**
HRmax	136.1 (7.36)	131.8 (8.96)	5.329***
MMSE	28.96 (1.09)	28.59 (1.35)	3.326**
BDI	6.06 (5.85)	5.22 (5.8)	1.576
Work ability			
WAI1: current work ability compared with the lifetime best	8.19 (1.45)	7.89 (1.53)	8.539
WAI2: work ability in relation to the demands of the job	8.64 (1.3)	8.21 (1.3)	14.753*
WAI3: number of current diseases diagnosed by a physician	4.28 (2.07)	2.73 (1.85)	73.696***
WAI4: estimated work impairment due to diseases	3.75 (0.58)	3.38 (0.88)	31.784***
WAI5: sick leave during the past year (12 months)	4.08 (0.87)	4.1 (0.9)	1.894
WAI6: own prognosis of work ability 2 years from now	6.93 (0.46)	6.83 (0.69)	3.028
WAI7: mental resources	3.14 (0.73)	3.28 (0.73)	5.048
Social life			
FriendsN	2.14 (0.87)	2.29 (0.91)	8.297*
FriendsFreq	2.65 (1.26)	2.13 (1.1)	26.575***
QoLsoc	15.2 (2.82)	14.88 (2.89)	1.24
Cognitive functions			
Stroop	− 9.11 (4.67)	− 12.44 (5.12)	7.06***
d2	170.56 (36.3)	144.05 (29.77)	8.7***
DST	65.24 (10.67)	55.62 (10.36)	9.63***
DS	14.49 (3.49)	13.83 (3.45)	2.05*
LPS3	30.02 (4.66)	26.18 (5.11)	8.26***
LPS7	23.7 (7.38)	20.78 (7.44)	4.16***
Physical fitness			
PWR	1.61 (0.45)	1.61 (0.49)	0.06
Pmax	100.82 (33.21)	109.68 (35.4)	− 2.61**
LPAQ	15.94 (8.87)	16.89 (8.97)	− 1.12

*BDI* beck depression inventory, *BMI* body mass index, *d2* d2-R test of attentional endurance, *DS* digit span backward, *DST*  digit substitution test, *FriendsFreq* frequency of social contacts, *FriendsN*   number of friends, *LPS3* logical reasoning,* LPS7*   spatial rotation, *LPAQ* lüdenscheid physical activity questionnaire, *MMSE* Mini-Mental State Examination, *Stroop* stroop test, *Pmax*  maximum power output in watt of the physical work capacity test; *PWR* power-to-weight ratio of the physical work capacity test; *QoLsoc* WHOQoL social dimension, *WAI* work ability index, *WHR* waist–hip ratio. * < 0.05; ** < 0.01; *** < 0.001

Concerning the variables used in the structural equation model, older adults informed of more current diseases (WAI3) and higher work impairment due to diseases (WAI4). They also rated their WA in relation to the job demands (WAI2) lower than their middle-aged counterparts. On the other hand, older adults informed of having more friends (FriendsN), while middle-aged adults had more frequent social contacts per week (FriendsFreq). Furthermore, older adults scored lower than middle-aged adults in all cognitive variables (Stroop, Digit-Symbol, d2-R, Digit-Span Backward, LPS3, and LPS7), but achieved a higher maximum power output (Pmax) on the bicycle ergometer test than the younger group.

### Correlations

**Fig. 2:**
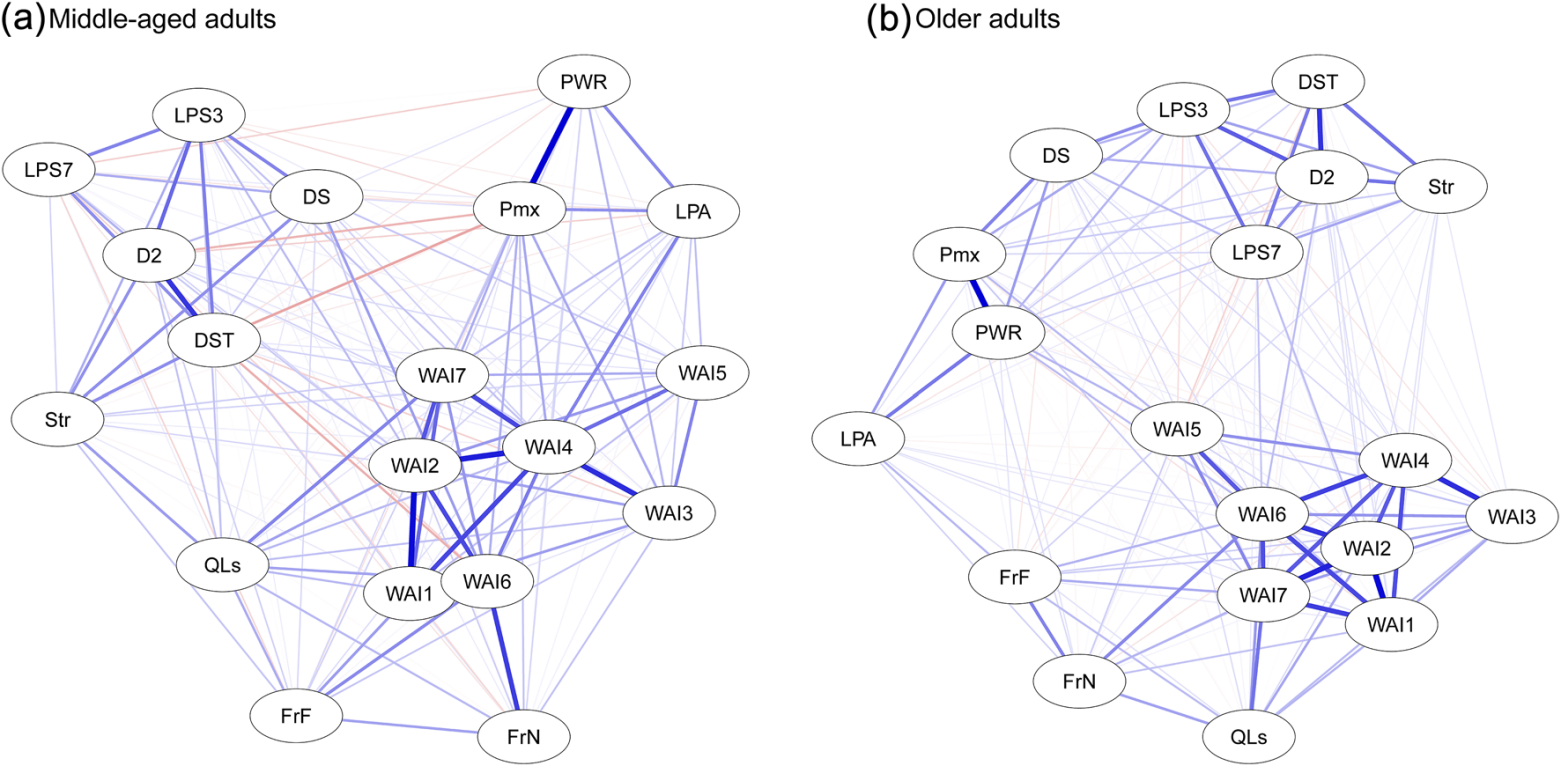
Network of associations based on polychoric, polyserial, and Pearson’s correlations for (**a**) middle-aged, and (**b**) older adults. Each node represents one of the indicator variables and the edges represent the strength of the correlations between the variables. Green edges represent positive correlations and red edges represent negative correlations. The width and color of the edges correspond to the absolute value of the correlations. Nodes are placed according to a forced-embedded algorithm (Fruchterman and Reingold 1991) in which the length of edges depends on the absolute weight of the edges. *d2*  d2-R Test of attentional endurance, *DS*  Digit-Span Backward, *DST*  Digit Substitution Test, *FrF*  frequency of social contacts, *FrN*  number of friends, *LPS3*  logical reasoning, *LPS7*  spatial rotation, *LPA*  lüdenscheid physical activity Questionnaire, *Str*  stroop test, *Pmx*  maximum power output in watt of the physical work capacity test; *PWR*  power-to-weight ratio of the physical work capacity test, *QLs*  WHOQoL social dimension, *WAI  *work ability index

Figure 2 depicts the correlational structures for each age group in a graphic network. Visual network analysis is a useful tool in exploratory data analysis for visualizing correlational structures within the data. In both groups, the indicator variables formed similar patterns, corresponding to the four hypothesized latent factors (Work Ability, Social Life, Fitness, and Cognitive Functions).

*Social Life vs. WA*. In both groups, the WHOQoL social dimension (QoLsoc) showed a positive relationship with current WA (WAI1), WA in relation to job demands (WAI2), fewer diseases (WAI3), and mental resources (WAI7). In middle-aged adults, more frequent social contacts (FriendsFreq) were associated with fewer diseases (WAI3), less work impairment due to diseases (WAI4), higher number of friends (FriendsN), and a more optimistic prognosis of WA (WAI6). In older adults, FriendsN was positively associated with current WA (WAI1), WA in relation to job demands (WAI2), the prognosis of WA (WAI6), and mental resources (WAI7). Furthermore, in this age group, FriendsFreq correlated with job demands (WAI2) and mental resources (WAI7), and QoLsoc with the prognosis of WA (WAI6).

*Cognitive Functions vs. WA.* In middle-aged adults, WA in relation to job demands (WAI2) was positively associated with processing speed (DST), logical reasoning (LPS3), selective attention (d2), and working memory (DS). Furthermore, in this age group, DS was related to fewer sick leaves (WAI5), and DST to more mental resources (WAI7). In older adults, LPS3 was associated with WA in relation to job demands (WAI2) and mental resources (WAI7), and no other significant correlations between cognitive and WAI variables were found in this age group.

*Physical Fitness vs. WA.* Overall, we found more associations between the fitness-related and the WAI variables in the group of middle-aged than in their older counterparts. In this sense, in middle-aged adults, the maximum power output (Pmax), the power-to-weight ratio (PWR), and the weekly activity level (LPAQ) were associated with more physical health (WAI3, WAI4, and WAI5). Furthermore, Pmax correlated in middle-aged adults positively with job demands (WAI2), and LPAQ with mental resources (WAI7). In older adults, only sick leaves (WAI5) were related to Pmax and PWR, and no other WAI variables showed a significant correlation with physical fitness in this age group.

### Structural equation modelling

#### Configural model and multi-group model fit

The posited model with the pooled data of both groups presented a regular fit $({\chi }^{2} = 347.435, df=146,p < 0.05, \mathrm{CFI }= 0.905,\mathrm{RMSEA }= .054, \mathrm{SRMR }= .07)$. An inspection of the modification indices indicated that linking the errors of WAI3 (number of current diseases diagnosed by a physician) and WAI4 (estimated work impairment due to diseases) would improve the model, reducing the standard ${\chi }^{2}$ by 50.815. Given that the estimation of work impairment is conditioned by the number of current diseases, it seemed reasonable to us to include this parameter in the model. After this adjustment, the model showed an acceptable fit to the pooled data (${\chi }^{2} = 300.529, df = 145, p < 0.05, \mathrm{CFI }= 0.927, \mathrm{RMSEA }= .047, \mathrm{SRMR }= .066)$. Factor loadings of all indicator variables were statistically significant (all $p\mathrm{s }< 0.001$) and no variable presented a negative error variance. The standardized parameter estimates indicated that WA was significantly explained by Social Life ($\gamma =.457, p<.001$) and Cognitive Functions ($\gamma = 0.172, p < 0.01$), while the path between WA and Physical Fitness was not significant ($\gamma = 0.088, p > 0.05$). Social Life was associated with Cognitive Functions ($r = 0.181, p < 0.05$), whereas Cognitive Functions and Physical Fitness, and Physical Fitness and Social Life were unrelated to each other ($p > 0 .05$).

In a second step, we tested for configural invariance in a multi-group approach. Therefore, we fitted the data of middle-aged and older adults simultaneously in the same model with Age Group as the grouping variable. As showed in Table 2, fit indices were adequate (${\chi }^{2} = 390.091, df = 290, p < 0.05, \mathrm{CFI }= 0.945, \mathrm{RMSEA }= 0 .038, \mathrm{SRMR }= 0 .079$) and no variable presented a negative error variance.

**Table 2:** Model fits and invariance tests across middle-aged and older adults

Model	${\chi }^{2}$	*df*	CFI	RMSEA	SRMR	Model comp	$\Delta {\chi }^{2}(\Delta df)$ ^a^	$\Delta$ CFI	Decision
M1: overall model	300.53	145	0.927	0.047	0.066	–	–	–	–
M2: configural model	390.09	290	0.945	0.038	0.079	–	–	–	–
M3: metric invariance	404.97	305	0.945	0.037	0.081	M2	14.88 (15)	0.000	Accept
M4: structural invariance	433.85	311	0.932	0.040	0.086	M3	28.88 (6)*	0.013	Reject
M5: structural invariance 2	405.44	309	0.947	0.036	0.081	M3	0.47 (4)	0	Accept

All fit indices are based on robust (scaled) statistics

Group 1: *n* = 236; group 2: *n* = 247; parameters: *n* = 78, *df*  degree of freedom; *CFI*  comparative fit index; *RMSEA* root-mean-square error of approximation; *SRMR* standardized root-mean-square residual

^a^Scaled Chi-squared difference test (method = “satorra.2000”)

In a third step, we tested for metric invariance, constraining the factor loadings to be equal across groups. Also, this model showed an adequate fit to the data of both groups (${\chi }^{2} = 404.972, df = 305,p < 0.05,\mathrm{CFI }= 0.945,\mathrm{RMSEA }= .037, \mathrm{SRMR }= 0 .081, \Delta {\chi }^{2} = 14.88,$ n.s.), indicating that latent constructs were defined similarly by both groups. Detailed information on factor loadings and parameter estimates of the different models are given in Online Resource Tables S2, S3, and S4.

#### Regressions and correlations across groups

**Fig. 3:**
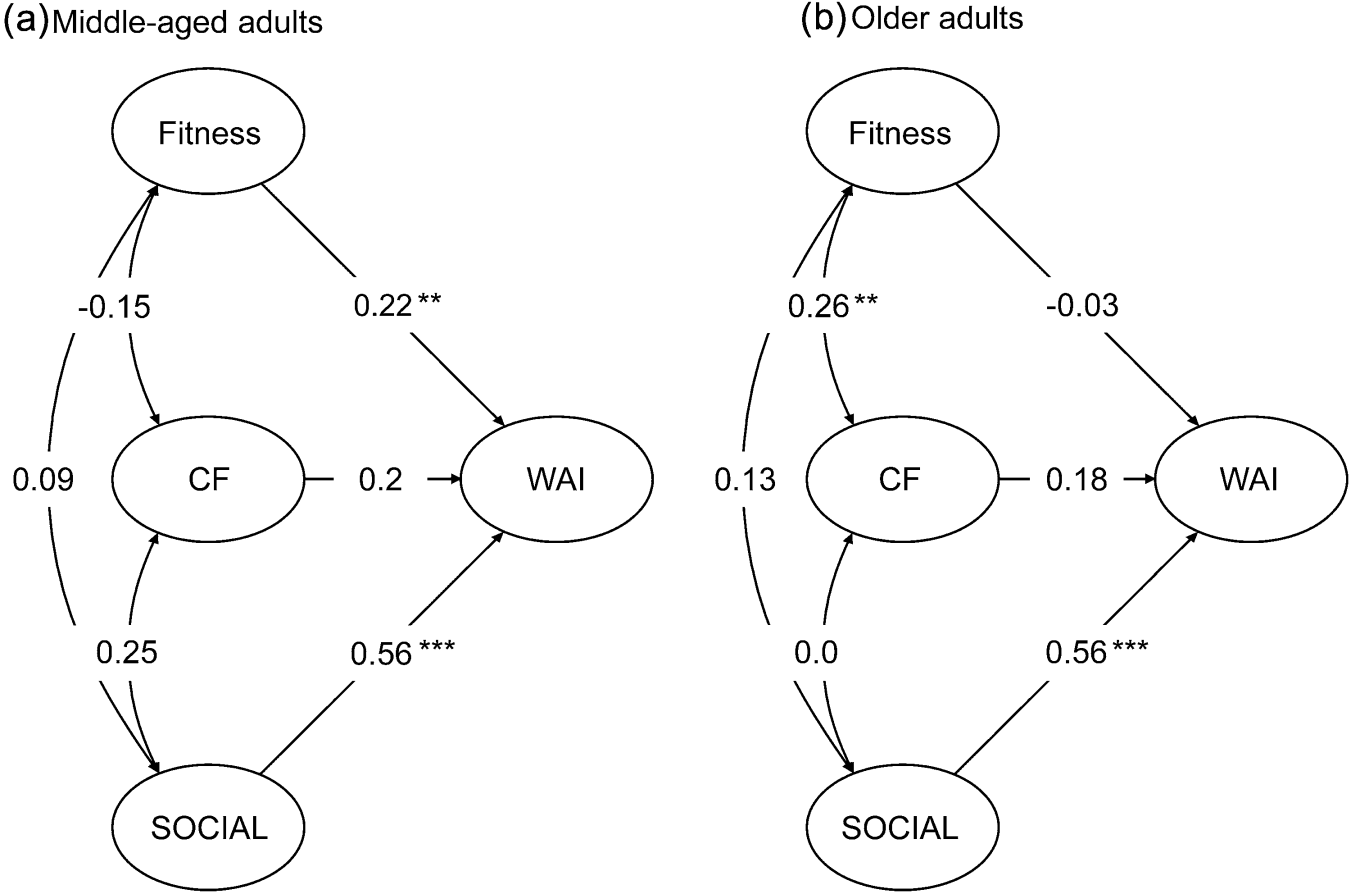
Path diagram for (**a**) middle-aged, and (**b**) older adults. Social, CF, and Fitness constitute the independent (exogenous) factors, and WAI, the dependent (endogenous factor). One-headed lines represent unstandardized regression coefficients, and two-headed curved lines represent correlations. *Social*  social life, *CF*  cognitive functions, *WAI* work ability index. Observed variables are omitted. * < .05; ** < .01; *** < .001

Figure 3 shows the partial regression coefficients and correlations between latent factors of the metric model for each age group. As indicated by the unstandardized parameter estimates, the path from Social Life to WA constituted the strongest predictor for WA in middle-aged adults ($$\gamma = 0.556, \mathrm{SE} = 0.196, p < 0.01)$$, as well as in older adults ($$\gamma =.558, SE=.16,p < 0.001$$). Contrary to the analysis with the pooled data, the path from Cognitive Functions to WA did not reach statistical significance neither in the group of middle-aged ($$\gamma = 0.196, \mathrm{SE} = 0.127, p > 0.05),$$ nor in the group of older adults $$(\gamma = 0176,\mathrm{SE} = 0.112, p > 0.05)$$. In middle-aged adults, Physical Fitness positively predicted WA ($$\gamma = 0.223,\mathrm{ SE }= 0.084, p < 0.01)$$, whereas in older adults, Fitness had no influence on WA ($$\gamma = -0.032,\mathrm{SE }= 0.073, p > 0.05)$$. Regarding the correlations between the latent factors, in older adults, Physical Fitness correlated significantly with Cognitive Functions ($$r = 0.259, p < 0.01)$$, whereas in middle-aged adults, Cognitive Functions were marginally associated with Social Life ($$r = 0.253, p = 0.05)$$.

In a last step, we tested for the statistical significance of group differences in these parameters. First, we set latent covariances and regression coefficients to be equal across groups, while maintaining the constraints on loadings. This model showed a significantly poorer fit in comparison to the unconstrained model ($\Delta {\chi }^{2} = 28.88 , p < .05$), confirming the existence of substantial differences in structural parameters across groups. Several *t* tests on the partial regression coefficients and on the correlations between latent factors revealed that the influence of Physical Fitness on WA was significantly larger in middle-aged than in older adults $(t = 2.304, p < .05)$. Social Life showed a stronger relationship with Cognitive Functions in middle-aged than in older adults $(t = 4.321, p < 0.01),$ whereas the correlation between Cognitive Functions and Fitness was significantly larger in older than in middle-aged adults $(t = 6.26, p < 0.01)$. None of the remaining structural parameters resulted statistically different across groups (all *p*s $< 0.05$). In a last step, to analyze how these differences influenced the model fit, we generated one more model, with latent covariances and regression coefficients constrained across groups, except for the path from Fitness to WA and the covariance between Fitness and Cognitive Functions. This model did not differ statistically from the metric model$(\Delta {\chi }^{2} = 0.47, n.s.)$, confirming that these were the only parameters whose group differences negatively affected the model fit.

## Discussion

The main aim of this study was to evaluate the influence of physical fitness, social life, and cognitive functions on WA in older and middle-aged adults, and to analyze the relationships between these constructs across groups. To this end, we proposed a model with three latent independent (exogenous) factors (Physical Fitness, Cognitive Functions and Social Life) and WA as dependent (endogenous) factor and compared the outcomes between both groups of participants. 

Regarding hypothesis 1, our results showed that Social Life was the strongest predictor for WA in both groups, indicating an age-independent positive effect of an active social life on WA. On the other hand, our data did not confirm hypothesis 2, as the contribution of Cognitive Functions to WA did not reach statistical significance in either group. Contrary to hypothesis 3, Physical Fitness was significantly associated with WA only in middle-aged adults. Regarding hypothesis 4, in older adults, Cognitive Functions correlated positively with Physical Fitness, whereas no such association was found in middle-aged adults. Hypothesis 5 could only be partially confirmed, as the correlation between Social Life and Physical Fitness did not reach statistical significance in either group. However, a comparison of the correlation indices across groups revealed a significantly stronger association between Cognitive Functions and Social Life in middle-aged workers than in the older group. Finally, we did not find a significant association between Social Life and Physical Fitness in either group and thus could not confirm hypothesis 6. 

Our results are in line with the growing evidence on the positive effect of social support outside the workplace on WA (Brady et al. 2020; Gould et al. 2008; Peters et al. 2018). Positive social support and good social functioning are related to fewer mental health issues, more adaptive coping strategies, and higher resilience to stress (Ozbay et al. 2007), which could help workers successfully fulfilling their work roles.

On the other hand, the positive association found between Physical Fitness and WA in middle-aged adults is in accordance with the existing literature about the beneficial effects of weekly leisure-time physical activity on WA (Airila et al. 2012; Grabara et al. 2018; Mohammadi et al. 2014; van den Berg et al. 2008b). However, in our study, the influence of fitness on WA was not present in older adults, suggesting the existence of age-dependent differences in how physical exercise is related to WA. Older adults had a similar fitness level as their younger counterparts, and even reached higher levels of maximum power output on the ergometer test. One possible explanation could be that with increasing age, people might get more concerned about their physical health and intend to raise their fitness level as a prevention for a health-related decline. In addition, it could be that older participants regarded the fitness test more as a challenge and therefore exerted themselves more than younger ones. The initial correlational analysis of the indicator variables had shown in middle-aged adults an association between the results of the ergometer test and the number of currently diagnosed diseases, which was not the case in older adults. Thus, one hypothetical reason for the different regression coefficients of the SEM model could be that the type of diseases experienced by older adults that are in good physical shape might be related to an age-related biological decline in health aspects that cannot be prevented with physical exercise. It remains unclear whether the influence of physical exercise on WA increases in physically lower performing older workers.

Regarding the influence of cognitive functions on WA, our results showed a small effect that did not reach statistical significance in either age group. Even though older adults scored lower in the cognitive indicator variables, cognition as a latent factor contributed to a similar degree to WA in both groups. As mentioned in the introduction, biological aging comes along with a decline in a series of cognitive functions, especially those involved in fluid abilities, such as reasoning, memory, or processing speed (Salthouse 2012). Given that the sample was composed of workers with predominantly mental work demands, this result suggests that older adults might compensate for a decline in cognitive abilities with increasing automaticity of cognitively demanding work tasks. In consonance with the previously discussed literature, we found a significant positive correlation between Physical Fitness and Cognitive Functions only in the group of older adults. As the influence of cognition on WA was similar in both groups despite older adults’ poorer cognitive performance, older workers might counterbalance cognitive declines with higher fitness levels. This interpretation would be in consonance with the existing evidence on the beneficial effects of fitness on cognition in older adults (Ballesteros et al. 2015; Gajewski and Falkenstein 2015, 2018; Kardys et al. 2022; Rieker et al. 2022). For example, Voelcker-Rehage et al. (2011) found in a 12-month intervention study that 3 days of cardiovascular or coordination training per week not only improved executive functions and processing speed, but also correlated with a reduction of prefrontal overactivation. Another study (Liu-Ambrose et al. 2010, 2012) found that 12-month resistance training produced significantly more improvements in executive functions than toning and balance training. Interestingly, the intervention did not detain the course of brain volume reduction, suggesting that cognitive improvements were more related to functional than structural training-induced brain changes. Finally, Social Life showed in middle-aged adults a significantly stronger association with Cognitive Functions than it was the case in older adults. The influence of social support on cognition has been widely investigated in older adults (Evans et al. 2019; Kelly et al. 2017), but less in healthy younger adults, for which it is difficult to interpret this finding. Furthermore, the correlation itself did not reach statistical significance and thus should be interpreted with caution. 

In sum, our results suggest that social life outside work might contribute to WA independently of the age of the worker. However, younger and older employees differed in the estimated relationship between physical activity and WA: whereas physical fitness played an important role for WA in middle-aged adults, this was not the case in older employees. Finally, cognitive functions seem to play a less important role for WA in middle-aged and older adults. This may be partly due to specific physical and work-related components captured by the WAI that do not share variance with the measures of cognitive functions. Alternatively, traditional psychometric tests are not diagnostically conclusive with respect to work-related questions and more ecologically valid instruments should be used to assess cognition in working life. 

## Limitations

There are some limitations that need to be considered when interpreting the present results. The first limitation refers to the cross-sectional data that do not allow for drawing causal conclusions. While the causal directions proposed and tested in the current study are consistent with findings of prior laboratory experiments, different research methodologies (e.g., longitudinal field research) would be beneficial to confirm the invariance of the findings of this study. It should be noted here that the Dortmund Vital Study is designed as a longitudinal study, which will facilitate such an analysis in the future. Second, our findings may not be generalizable to the general working population as the sample was mainly composed of white-collar workers with a predominantly mental workload. It remains unclear if the influence of fitness, cognition, and social life on WA is modulated by the type of work a person performs.

Finally, while cognitive functions and physical fitness were assessed by objective measurements, some of the variables included in this study are based on self-reporting tools that are associated with limitations such as inaccurate reporting and social desirability bias and might lead to biased estimates of model relationships.

## Concluding remarks

According to the results of the study, WA seems to be affected by different factors over the working lifespan. In particular, social activities outside the work and leisure-time physical exercise increase well-being and health and may act as a catalyst for work-associated negative aspects like psychosocial stress or even low work conditions.

## Supplementary Information

Below is the link to the electronic supplementary material.

Supplementary file1 (PDF 1007 KB)

## Data Availability

The datasets and codes generated during and/or analyzed during the current study are available from the corresponding author on reasonable request.
