# Supplementary material for: The impact of physical fitness, social life, and cognitive functions on work ability in middle-aged and older adults
Source: Int Arch Occup Environ Health. 2022 Dec 16;96(4):507–20. doi: 10.1007/s00420-022-01943-8 (PMC10079745; doi:10.1007/s00420-022-01943-8)
Supplement: Supplementary file 1 — Supplementary file1 (PDF 1007 KB) [file 420_2022_1943_MOESM1_ESM.pdf]

SUPPLEMENTARY FILES

The impact of physical fitness, social life, and cognitive functions on work ability in middle-aged and older adults

Jennifer A. Rieker, PhD[1], Patrick D. Gajewski, PhD[2], José Manuel Reales, PhD [3], Soledad Ballesteros, PhD [1], Klaus Golka, PhD [2], Jan G. Hengstler, PhD [2], Edmund Wascher, PhD [2], Stephan Getzmann, PhD [2]

[1]Department of Basic Psychology II, Universidad Nacional de Educación a Distancia, Madrid, Spain

[2]Leibniz Research Centre for Working Environment and Human Factors at TU Dortmund (IfADo), Dortmund, Germany

[3]Department of Methodology for Behavioral Science, Universidad Nacional de Educación a Distancia, Madrid, Spain

* Corresponding author:

Jennifer A. Rieker
Department of Basic Psychology II,
Universidad Nacional de Educación a Distancia,
Juan del Rosal, 10, 28040 Madrid, Spain
Email: jrieker@psi.uned.es

Table S1. Sociodemographic and lifestyle characteristics of the sample as a function of the WAI score.

**Characteristics**	**N (%)**	**WAI (SD)**	***F***	***p***
Age groups[a]			27.25	<.001
middle-aged adults	247 (51.1%)	40.7 (6.2)		
older adults	236 (48.9%)	38.2 (5.3)		
Family and social relationships				
Marital status			< 1	ns.
single	36 (7.5%)	40.3 (5.1)		
married	181 (37.9%)	39.4 (5.7)		
partnership	220 (46.0%)	39.4 (5.4)		
divorced	35 (7.3%)	39.7 (5.1)		
widowed	6 (1.3%)	39.3 (4.1)		
Living situation			2.3	ns.
alone	102 (22.9%)	39.4 (6.2)		
with partner	138 (31%)	39.5 (4.9)		
with family	154 (34.6%)	38.9 (5.5)		
with parents	8 (1.8%)	43.2 (2.3)		
with other persons	43 (9.7%)	41.0 (4.6)		
Children			2.0	ns.
no	325 (67.6%)	39.7 (5.3)		
yes	156 (32.4%)	38.9 (5.5)		
Family pet			4.3	.039
no	379 (78.8%)	39.7 (5.2)		
yes	102 (21.2%)	38.5 (5.8)		
Number of close friends			2.2	ns.
1 - 3	97 (21.2%)	38.3 (5.6)		
4 - 7	222 (48.5%)	39.4 (5.3)		
8 - 10	88 (19.2%)	40.9 (5.0)		
more than 10	51 (11.1%)	40.3 (5.0)		
Frequency of friendship meetings			4.1	.003
once or twice per month	136 (28.5%)	38.1 (5.7)		
once per week	140 (29.3%)	39.4 (5.4)		
twice per week	110 (23%)	40.1 (4.9)		
three times per week	62 (13%)	41.1 (5.0)		
more than three times per week	30 (6.3%)	40.5 (4.7)		
Nutrition and stimulants				
Frequency of *fruit* consumption			1.35	ns.
several times a day	79 (16.5%)	39.2 (5.4)		
daily	182 (37.9%)	40.0 (5.5)		
one to four times per week	197 (41%)	39.2 (5.0)		
once per month	18 (3.8%)	38.1 (6.1)		
never	4 (0.8%)	37.8 (5.9)		

Frequency of *vegetable* consumption			< 1	ns.
daily	70 (14.6%)	40.2 (5.6)		
one to four times per week	218 (45.4%)	39.7 (5.4)		
once per month	186 (38.8%)	38.9 (5.2)		
never	6 (1.3%)	38.0 (5.8)		
Frequency of *wholemeal* consumption			< 1	ns.
several times a day	36 (7.5%)	40.3 (5.1)		
daily	181 (37.9%)	39.4 (5.7)		
one to four times per week	220 (46%)	39.4 (5.4)		
once per month	35 (7.3%)	39.7 (4.1)		
never	6 (1.3%)	39.3 (4.1)		
Frequency of *meat* consumption			< 1	ns.
several times a day	92 (19.2%)	39.5 (5.5)		
daily	110 (23%)	39.8 (4.9)		
one to four times per week	196 (40.9%)	39.3 (5.5)		
once per month	28 (5.8%)	38.7 (5.9)		
never	53 (11.9%)	39.7(5.3)		
Frequency of *fish* consumption			1.74	ns.
several times a day	2 (12.3%)	46.0 (.0)		
daily	4 (39.3%)	41.7 (2.2)		
one to four times per week	226 (46.8%)	39.7 (5.3)		
once per month	190 (39.5%)	38.0 (5.5)		
never	59 (12.3%)	39.5 (5.3)		
Frequency of *fast-food* consumption			1.29	ns.
several times a day	1 (.2%)	40.0 (.0)		
daily	3 (.6%)	38.6 (1.5)		
one to four times per week	120 (25%)	39.1 (5.8)		
once per month	301 (62.7%)	38.9 (5.6)		
never	55 (11.5%)	40.6 (5.4)		
Frequency of *alcohol* consumption			1.58	ns.
daily	24 (5%)	38.1 (6.0)		
one to three times per week	171 (35.6%)	39.0 (5.6)		
one to three times per month	220 (45.8%)	39.7 (5.3)		
never	65 (13.5%)	40.3 (4.6)		
Frequency of *smoking*			< 1	ns.
more than 20 per day	9 (1.9%)	37.9 (6.6)		
11 – 20 per day	22 (4.6%)	40.0 (5.7)		
1 – 10 per day	43 (9%)	40.2 (4.7)		
not anymore	103 (21.5%)	39.4 (5.0)		
never	302 (63%)	39.4 (5.5)		
Education and Lifestyle				
Education			6.73	<.0001
university degree	213 (44.5%)	40.3 (5.1)		

high school diploma	151 (31.5%)	39.2 (5.2)		
secondary	94 (19.6%)	37.5 (5.6)		
primary	21 (4.4%)	37.9 (5.9)		
Frequency of using a foreign language			6.85	<.0001
often	143 (29.9%)	40.7 (4.8)		
sometimes	157 (32.8%)	39.2 (4.9)		
rarely	143 (29.9%)	39.1 (5.8)		
never	36 (7.5%)	36.5 (6.7)		
Sleep quality			20.55	<.0001
always good	44 (9.1%)	41.9 (4.4)		
mostly good	299 (62.2%)	40.3 (4.7)		
rarely good	131 (27.2%)	36.9 (6.0)		
never good	7 (1.5%)	34.0 (5.3)		
Watching TV			7.22	<.0001
1 hour or less per day	63 (22.4%)	40.9 (4.8)		
1 – 2 hours per day	127 (45.2%)	38.3 (5.6)		
2 – 3 hours per day	65 (23.1%)	40.7 (4.1)		
more than 3 hours per day	26 (9.3%)	36.8 (5.0)		
PC using			< 1	ns.
1 hour or less per day	91 (46%)	38.9 (5.9)		
1 – 2 hours per day	64 (32.3%)	39.7 (4.2)		
2 – 3 hours per day	26 (13.1%)	40.0 (4.8)		
more than 3 hours per day	17 (8.6%)	38.5 (4.8)		
Mobile phone using			< 1	ns.
1 hour or less per day	121 (52.6%)	39.6 (5.1)		
1 – 2 hours per day	69 (30%)	39.2 (5.9)		
2 – 3 hours per day	21 (9.1%)	39.5 (5.0)		
more than 3 hours per day	19 (8.3%)	39.2 (4.3)		
Media general			2.08	ns.
1 hour or less per day	46 (11.6%)	38.6 (6.2)		
1 – 2 hours per day	80 (20.2%)	39.8 (6.0)		
2 – 3 hours per day	97 (24.4%)	40.3 (4.0)		
more than 3 hours per day	174 (43.8%)	38.8 (5.2)		
Duration of physical activity			3.06	.028
1 hour or less per week	46 (12.9%)	39.5 (4.9)		
1 – 2 hours per week	96 (26.9%)	38.7 (5.4)		
2 – 3 hours per week	59 (16.5%)	39.5 (5.4)		
more than 3 hours per week	156 (43.7%)	40.6 (4.9)		
Work-related parameters				
Work amount			2.01	ns.
full position (100%)	217 (47.8%)	39.7 (5.5)		
three-quarter position (75%)	63 (13.9%)	38.0 (5.3)		
half position (50%)	57 (12.6%)	38.9 (4.9)		

less than 50%	37 (8.1%)	39.8 (4.9)		
mini job	80 (17.6%)	40.3 (5.3)		
Employment sector			4.98	.002
industry	38 (8.4%)	39.1 (5.5)		
service	296 (65.2%)	38.8 (5.6)		
education	111 (24.4%)	41.0 (4.4)		
craft	9 (2%)	39.2 (4.6)		
Type of work			3.28	ns.
repetitive	45 (9.3%)	38.1 (5.3)		
flexible	409 (84.7%)	39.6 (5.3)		
Shift work			1.41	ns.
no shift work (day)	340 (70.4%)	39.5 (5.4)		
shift work without night work	5 (1.2%)	38.4 (11.1)		
shift work with night work	37 (8.9%)	39.8 (5.3)		
night work only	33 (8%)	37.1 (5.3)		
Commuting to work			1.04	ns.
1 hour or less per day	260 (75.1%)	39.1 (5.6)		
1 – 2 hours per day	69 (19.9%)	39.2 (5.4)		
2 – 3 hours per day	12 (3.5%)	41.2 (4.0)		
more than 3 hours per day	5 (1.4%)	42.2 (2.9)		

[a]Age groups are defined as followed: middle-aged = 20 to 44 years; older = 45 years or older

Table S2 Factor loadings and parameter estimates of the structural equation model for the whole sample

Latent factors and indicator variables	*B*	ci.lower	ci.upper	*b*	R^2
Social					
QoLsoc	1.000	1.000	1.000	0.607	0.369
FriendsN	0.559	0.285	0.833	0.339	0.115
FriendsFreq	0.748	0.414	1.082	0.453	0.206
**Cognitive Functions**					
DST	1.000	1.000	1.000	0.722	0.521
LPS3	0.929	0.756	1.102	0.671	0.450
Stroop	0.773	0.621	0.926	0.559	0.312
d2	1.008	0.843	1.173	0.728	0.530
LPS7	0.670	0.517	0.822	0.484	0.234
DS	0.506	0.352	0.660	0.366	0.134
**Physical Fitness**					
Pmax	1.000	1.000	1.000	0.787	0.620
PWR	1.064	0.752	1.376	0.838	0.702
LPAQ	0.515	0.352	0.679	0.406	0.165
WAI					
WAI1	1.000	1.000	1.000	0.788	0.622
WAI2	1.088	0.982	1.195	0.858	0.736
WAI6	0.918	0.763	1.074	0.724	0.524
WAI7	0.750	0.648	0.852	0.591	0.350
WAI3	0.493	0.371	0.615	0.389	0.151
WAI5	0.380	0.250	0.511	0.300	0.090
WAI4	0.846	0.736	0.956	0.667	0.445
**Regressions**					
WAI ~Fitness	0.088	-0.022	0.199	0.088	
WAI ~Social	0.594	0.300	0.887	0.457	
WAI ~CF	0.188	0.063	0.312	0.172	
**Covariances/ Correlations**					
WAI3 ~~WAI4	0.363	0.271	0.455	0.528	
Social ~~CF	0.079	0.013	0.145	0.181	
Social ~~Fitness	0.045	-0.029	0.119	0.094	
CF ~~Fitness	0.008	-0.059	0.075	0.014	

B = unstandardized estimate; *b* = standardized estimate; ci = confidence interval; R^2 = R square; d2 = d2-R Test of attentional endurance; DS= Digit Span Backward; DST = Digit Substitution Test; FriendsFreq = Frequency of social contacts; FriendsN = Number of friends; LPS3 = logical reasoning; LPS7 = spatial rotation; LPAQ = Lüdenscheid Physical Activity Questionnaire; Pmax= maximum power output in watt of the Physical Work Capacity test; PWR = power-to-weight ratio of the Physical Work Capacity test; QoLsoc = WHOQoL social dimension; Stroop = Stroop Test; WAI = Work Ability Index

Table S3 Factor loadings and parameter estimates of the configural model for middle-aged and older adults.

	Middle-aged adults					*Older adults*				
**Latent factors and indicator variables**	***B***	**ci.lower**	**ci.upper**	***b***	R^2	***B***	**ci.lower**	**ci.upper**	***b***	R^2
Social										
QoLsoc	1.000	1.000	1.000	0.678	0.460	1.000	1.000	1.000	0.580	0.336
FriendsN	0.432	0.068	0.797	0.289	0.084	0.885	0.460	1.310	0.518	0.268
FriendsFreq	0.567	0.171	0.964	0.379	0.144	0.749	0.309	1.190	0.439	0.193
**Cognitive Functions**										
DST	1.000	1.000	1.000	0.622	0.387	1.000	1.000	1.000	0.677	0.458
LPS3	0.922	0.632	1.213	0.596	0.356	1.012	0.707	1.317	0.630	0.397
Stroop	0.727	0.482	0.972	0.462	0.213	0.809	0.546	1.072	0.495	0.245
d2	1.184	0.856	1.513	0.670	0.449	0.956	0.722	1.190	0.696	0.485
LPS7	0.646	0.379	0.914	0.379	0.144	0.827	0.594	1.060	0.510	0.260
DS	0.629	0.348	0.909	0.360	0.130	0.625	0.350	0.900	0.383	0.146
**Physical Fitness**										
Pmax	1.000	1.000	1.000	0.870	0.757	1.000	1.000	1.000	0.816	0.666
PWR	0.798	0.492	1.104	0.691	0.478	1.089	0.653	1.524	0.877	0.770
LPAQ	0.520	0.264	0.775	0.438	0.192	0.446	0.215	0.676	0.371	0.138
WAI										
WAI1	1.000	1.000	1.000	0.771	0.594	1.000	1.000	1.000	0.805	0.648
WAI2	1.097	0.968	1.226	0.846	0.715	1.045	0.903	1.187	0.841	0.707
WAI6	0.866	0.634	1.098	0.668	0.446	0.928	0.714	1.142	0.747	0.558
WAI7	0.738	0.598	0.879	0.569	0.324	0.932	0.804	1.059	0.750	0.562
WAI3	0.479	0.305	0.653	0.369	0.136	0.405	0.229	0.581	0.326	0.106
WAI5	0.454	0.287	0.621	0.350	0.122	0.376	0.198	0.554	0.303	0.092
WAI4	0.963	0.795	1.131	0.742	0.551	0.736	0.593	0.879	0.592	0.351
**Regressions**										
WAI ~Fitness	0.238	0.074	0.403	0.258		-0.043	-0.196	0.109	-0.045	
WAI ~Social	0.459	0.070	0.848	0.398		0.648	0.282	1.013	0.471	
WAI ~CF	0.194	-0.058	0.447	0.144		0.184	-0.044	0.412	0.138	
**Covariances/ Correlations**										
WAI3 ~~WAI4	0.275	0.122	0.429	0.442		0.388	0.257	0.518	0.509	
Social ~~CF	0.107	0.019	0.195	0.280		0.001	-0.068	0.071	0.004	
Social ~~Fitness	0.040	-0.079	0.160	0.072		0.065	-0.038	0.169	0.134	
CF ~~Fitness	-0.075	-0.162	0.011	-0.158		0.132	0.035	0.229	0.261	

B = unstandardized estimate; *b* = standardized estimate; ci = confidence interval; R^2 = R square; d2 = d2-R Test of attentional endurance; DS= Digit Span Backward; DST = Digit Substitution Test; FriendsFreq = Frequency of social contacts; FriendsN = Number of friends; LPS3 = logical reasoning; LPS7 = spatial rotation; LPAQ = Lüdenscheid Physical Activity Questionnaire; Pmax= maximum power output in watt of the Physical Work Capacity test; PWR = power-to-weight ratio of the Physical Work Capacity test; QoLsoc = WHOQoL social dimension; Stroop = Stroop Test; WAI = Work Ability Index

Table S4 Factor loadings and parameter estimates of the metric model for middle-aged and older adults. Factor loadings are constrained across groups.

	Pooled estimates				***Middle-aged adults***		***Older adults***	
**Latent factors and indicator variables**	***B***	**ci.lower**	**ci.upper**		***b***	R^2	***b***	R^2
Social								
QoLsoc	1.000	1.000	1.000		0.596	0.355	0.652	0.424
FriendsN	0.668	0.393	0.943		0.392	0.154	0.440	0.193
FriendsFreq	0.649	0.355	0.943		0.381	0.145	0.427	0.183
**Cognitive Functions**								
DST	1.000	1.000	1.000		0.615	0.378	0.678	0.460
LPS3	0.973	0.761	1.186		0.622	0.387	0.608	0.369
Stroop	0.765	0.587	0.943		0.480	0.230	0.469	0.220
d2	1.047	0.847	1.247		0.586	0.343	0.765	0.586
LPS7	0.748	0.571	0.925		0.433	0.188	0.462	0.214
DS	0.634	0.436	0.832		0.359	0.129	0.389	0.151
**Physical Fitness**								
Pmax	1.000	1.000	1.000		0.843	0.711	0.859	0.738
PWR	0.920	0.664	1.175		0.772	0.597	0.780	0.608
LPAQ	0.472	0.301	0.642		0.385	0.148	0.413	0.171
WAI								
WAI1	1.000	1.000	1.000		0.776	0.602	0.795	0.633
WAI2	1.081	0.981	1.182		0.839	0.704	0.860	0.740
WAI6	0.905	0.748	1.063		0.703	0.494	0.720	0.519
WAI7	0.848	0.754	0.941		0.658	0.433	0.674	0.455
WAI3	0.438	0.314	0.562		0.340	0.116	0.349	0.122
WAI5	0.414	0.291	0.536		0.321	0.103	0.329	0.108
WAI4	0.840	0.730	0.950		0.652	0.425	0.668	0.447
		Middle-aged adults				***Older adults***		
**Regressions**	***B***	**ci.lower**	**ci.upper**	***b***	***B***	**ci.lower**	**ci.upper**	***b***
WAI ~Fitness	0.223	0.061	0.385	0.232	-0.032	-0.175	0.112	-0.035
WAI ~Social	0.556	0.173	0.939	0.421	0.558	0.246	0.870	0.462
WAI ~CF	0.196	-0.053	0.445	0.143	0.176	-0.044	0.396	0.134
**Covariances/ Correlations**								
WAI3 ~~WAI4	0.328	0.183	0.472	0.460	0.348	0.219	0.476	0.499
Social ~~CF	0.084	0.000	0.168	0.253	0.001	-0.076	0.077	0.002
Social ~~Fitness	0.045	-0.061	0.150	0.094	0.074	-0.046	0.194	0.128
CF ~~Fitness	-0.068	-0.149	0.013	-0.148	0.138	0.038	0.238	0.259

B = unstandardized estimate; *b* = standardized estimate; ci = confidence interval; R^2 = R square; d2 = d2-R Test of attentional endurance; DS= Digit Span Backward; DST = Digit Substitution Test; FriendsFreq = Frequency of social contacts; FriendsN = Number of friends; LPS3 = logical reasoning; LPS7 = spatial rotation; LPAQ = Lüdenscheid Physical Activity Questionnaire; Pmax= maximum power output in watt of the Physical Work Capacity test; PWR = power-to-weight ratio of the Physical Work Capacity test; QoLsoc = WHOQoL social dimension; Stroop = Stroop Test; WAI = Work Ability Index
